# A Rare Case Report of Benign Intracranial Hypertension Caused by Hypervitaminosis A

**DOI:** 10.7759/cureus.59401

**Published:** 2024-04-30

**Authors:** Hanane Hajaj, Ayad Ghanam, Hind Zahiri, Abdeladim Babakhouya, Maria Rkain

**Affiliations:** 1 Pediatrics, Faculty of Medicine and Pharmacy, Mohammed I University of Oujda, Oujda, MAR; 2 Pediatrics, Centre Hospitalier Universitaire Mohammed VI, Oujda, MAR

**Keywords:** papilledema, cerebrospinal fluid, children, vitamin a, benign intracranial hypertension

## Abstract

Benign intracranial hypertension (BIH) in children is recognized as elevated intracranial pressure without hydrocephalus or intracranial mass. It manifests differently in adults, with no apparent predilection for sex or weight. Headache, papilledema, and possibly sixth nerve palsy with visual field defects are the typical symptoms of this syndrome. Vitamin A toxicity is a rare cause of BIH. We report the case of a previously healthy 13-year-old girl presenting with photophobia, a frontal headache, and vomiting. She had bilateral papilledema discovered by fundoscopy. Both magnetic resonance imaging and brain CT were normal. At admission, a lumbar puncture (LP) revealed an opening pressure of 26 cm H_2_O with normal cerebrospinal fluid (CSF) analysis. The diagnosis of BIH was established, and treatment with acetazolamide was started, with good clinical results. Regular eye evaluations showed a regression of papilledema. Elevated serum vitamin A levels were the only positive findings. Within two weeks, the patient was discharged without any symptoms.

This study aims to attract the attention of clinicians to the importance of evaluating vitamin A toxicity in the context of papilledema and oculomotor problems in a child who has undergone normal neuroradiological investigations.

## Introduction

Benign intracranial hypertension (BIH), also known as idiopathic intracranial hypertension (IIH) or pseudotumor cerebri (PTC), frequently affects adults. It is uncommon in children, and the diagnosis requires the exclusion of all other causes of intracranial hypertension [1]. Many hypotheses have been proposed to explain the etiology of BIH. The clinical syndrome consists of an elevation in intracranial pressure accompanied by papilledema in a person who is generally healthy. It usually disappears spontaneously, and its recurrence is uncommon. Most cases of BIH appear in women between the ages of 14 and 40. It has also been documented in obese adolescent females, in early pregnancy, after abortion, and in obese women in their 30s who suffer from irregular menstruation. The shared factor in these cases appears to be an unidentified hormonal disorder. Several other uncommon factors have been related to this syndrome, such as vitamin A toxicity [1]. Hypervitaminosis A has been associated with BIH in children; nevertheless, the physiopathology remains unknown. Diagnosis can easily be missed or delayed because the toxic symptoms of hypervitaminosis A appear so insidiously [2]. Here, we present an unusual case of a 13-year-old girl who was diagnosed with BIH due to hypervitaminosis A.

## Case presentation

**Figure 1:**
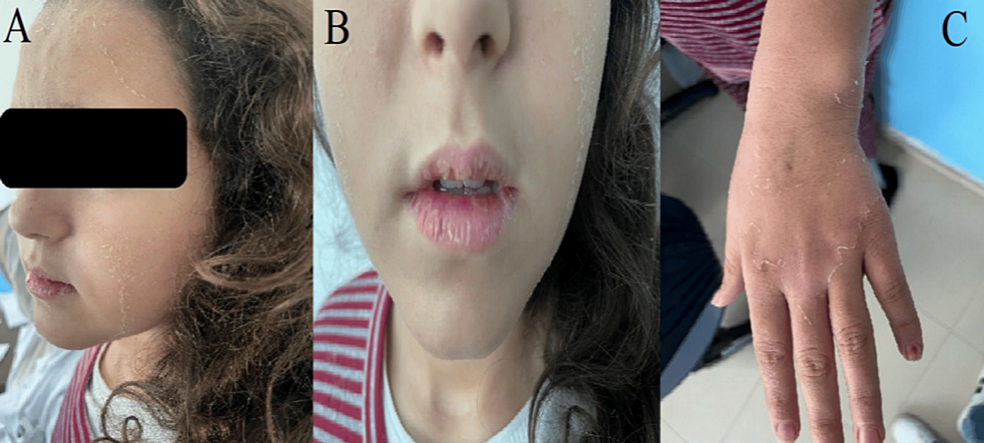
Clinical symptoms in our case (A and B) Scales on the face. (C) Scales on the left hand.

A 13-year-old girl, firstborn to a non-consanguineous couple, complained of intense frontal headaches, vomiting, and photophobia. These symptoms persisted for seven days. She has never experienced a trauma or similar eruption in her medical history. Additionally, she does not have a family history of illness. On admission, vital signs were normal. Quetelet index was 20.6 kg/m^2^ for a normal between 14.8 and 21.7 kg/m^2^. The neurological evaluation revealed a perceptive, cooperative, person-place-time-oriented patient. She also showed no signs of mental, verbal, or memory disorders. The skin was generally hyperemic, and she presented with a scaly rash associated with intense pruritus (Figure 1).

**Figure 2:**
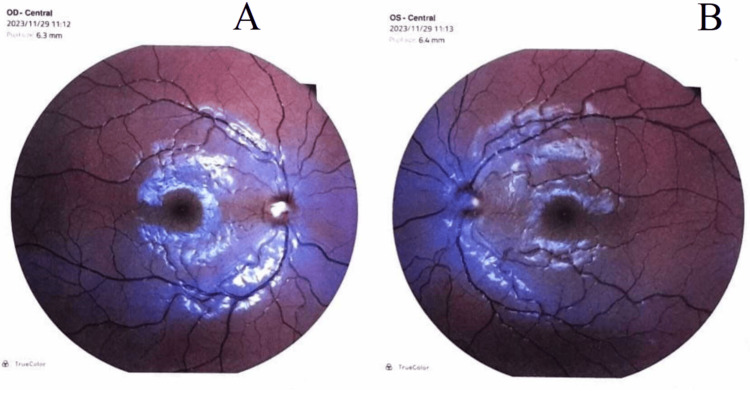
Bilateral papilledema: (A) right eye and (B) left eye

She had 10/10 visual acuity in both her left and right eyes. Funduscopic examination showed bilateral papilledema (stage 4), (Figure 2).

Exploration by magnetic resonance imaging (MRI) and brain CT showed no occupying space injury, venous thrombosis, or stenosis. All blood tests, including blood counts, erythrocyte sedimentation rate, C-reactive protein, and renal function, as well as liver blood tests, were normal (Table 1).

**Table 1:** Our patient's biological results at admission SGOT: Serum glutamic oxaloacetic transaminase; SGPT: Serum glutamic pyruvic transaminase.

Laboratory parameters	Initial values	Reference ranges
SGOT (UI/l)	14	5-34
SGPT (UI/l)	9	5-55
Urea (g/L)	0.27	0.10-0.30
Creatinine (mg/L)	5.9	5.7-11.1
Erythrocyte sedimentation rate (mm/hour)	8	0-20
C-reactive protein (mg/L)	1.63	0-5
Hemoglobin (g/dl)	14.7	12-16
White blood cell (/µ)	9100	4000-10000
Lymphocyte (/µl)	3690	2000-4000
Neutrophil (/µl)	4750	1500-7000

Cerebrospinal fluid (CSF) opening pressure in the lateral decubitus was 26 cm H_2_O, with normal analysis (Table 2).

**Table 2:** Cytobacteriological and biochemical analysis of CSF CSF: Cerebrospinal fluid.

Biological sample		
Appearance	Clear	
Color	Colorless	
Cytology		
White blood cells	0.00/mm³	
Red blood cells	0.00/mm³	
Microscopic examination after gram staining		
Microscopic examination after gram 1 staining	Absence of bacterial flora	
Research for soluble antigens		
Research for soluble antigens	Nothing to report	
Culture		
Culture 1	The culture remains sterile after 5 days of incubation	
Chemical examination		
Parameters	Results	Reference range
Glucose CSF	0.67 g/l	0. 60-0.80 g/l
Protein CSF	0.18 g/l	0.15-0.40 g/l
Chlore CSF	127 mmol/l	110.00-130.00 mmol/l

The medical history revealed the ingestion of a significant quantity of tuna liver. The dose of vitamin A (retinol) in her plasma seven days following consumption was elevated to 3.90 µmol/l (normal range: 0.91-2.51). The diagnosis of benign intracranial hypertension caused by vitamin A excess has been established based on all of these arguments. She was treated with 15 mg/kg/day of acetazolamide. Both eyes' papilledema had disappeared after two weeks of monitoring. According to this analysis, hypervitaminosis A was the only cause that could be incriminated.

## Discussion

BIH is a rare clinical entity due to an elevation of intracranial pressure in the absence of an organic cause that explains it. It is an uncommon condition affecting one in 100,000-150,000 children [3]. About 60% of children who present the syndrome are older than 10 years, and the frequency rises between the ages of 12 and 15 [4].

The most frequent clinical manifestation is headache, reported in 91% of cases; it is usually pulsating, intermittent, diffuse, and gets worse upon waking. Symptoms, including nausea and vomiting, are frequent. Tinnitus, photopsia, and retroocular pain may also occur. The most significant manifestation in children with BIH is papilledema. It is usually bilateral, but it can occasionally be unilateral or absent in newborns whose sutures have not fused. The diagnosis of BIH is not excluded, even if papilledema is absent. Reports indicate that 46%-60% of cases had sixth cranial nerve palsy. Visual acuity loss was documented in 6%-20% of cases, while visual field defects were observed in 91% of reports [5]. The syndrome of BIH most frequently affects young obese women, but it may also occur in males. Other causes have been associated with pseudotumor cerebri: medication-related (growth hormone and tetracyclines), vitamin A toxicity, hypoparathyroidism, Addison's disease, hypophosphatemia, and menstrual irregularity resulting from abnormal endocrine function [6].

The first case of BIH induced by vitamin A was described in 1856 by Elisha Kane, who reported vertigo and headache after consuming polar bear liver. Vitamin A can be found in many foods, such as liver, dairy products, egg yolks, fish, and yellow and green vegetables [7], and it has lots of uses, like the treatment of acne, fad diets, common cold prophylaxis, and the treatment of keratosis follicularis [6]. The recommended dietary allowance (RDA) of vitamin A changes according to age. Children aged one to three years should receive 400 μg retinol equivalents (RE) daily, with a maximum of 800 μg RE/day. Acute hypervitaminosis A has been reported with acute ingestion of 20 to 100 times the RDA [8]. In our case, the parents supplemented their daughter's diet with the liver of tuna rich in vitamin A, believing that this would strengthen her immune system. Few reports exist on the pathophysiology of intracranial hypertension caused by vitamin A. The exact pathophysiology of intracranial hypertension induced by hypervitaminosis A is unknown. It has been proposed that vitamin A metabolites reduce CSF reabsorption, potentially via regulating genes from all-trans-retinoic acid, which is mostly present in the choroid plexus and meninges [7].

The three primary organ systems impacted by vitamin A toxicity are the central nervous system, the skeletal/osseous system, and the integument. Central nervous system disorders consist of headaches, cranial nerve sixth palsies, and impaired vision, frequently accompanied by normal mental status. Skeletal abnormalities include cortical thickening accompanied by pain and tenderness at the site of bony involvement. Skin alterations generally develop with chronic toxicity involving lip fissures, alopecia, coarse hair, desquamation of the palms and soles, and pruritus. Nonspecific signs, including nausea, vomiting, and lethargy, may manifest in infants. Fundoscopy may identify papilledema, but the rest of the physical exam, including the neurologic findings, is usually normal. CT or MRI should be performed on all patients to rule out other possible reasons for elevated intracranial pressure. Radiological studies are usually normal. A lumbar puncture (LP) must be performed following a CT. Classically, the composition of the CSF is normal [6]. Opening pressure greater than 28 cm H_2_O in children qualifies as increased. Nevertheless, an opening pressure higher than 25 cm H_2_O is classified as abnormal in children who are not obese and who were not sedated during the LP [7]. The evaluation of vitamin A levels in people with toxicity may be complicated because serum retinol concentrations are nonsensitive markers.

The main goal of treatment is to prevent vision loss. There are no clinical trials for evidence-based recommendations in the management of pediatric pseudotumor cerebri. In the treatment of pediatric patients, acetazolamide is often utilized. The recommended initial dose is 15-25 mg/kg/day, administered in two to three divided doses each day. This can be raised progressively to 100 mg/kg but not more than 2 g/day for children and 4 g/day for adolescents. Alternatives like corticosteroids, topiramate, and furosemide can also be utilized. Treatment must be maintained until the papilledema resolves. The follow-up is founded on the visual evaluations, optic nerve appearance, and functional symptoms of increased intracranial pressure. Surgical procedures such as optic nerve sheath fenestration or cerebrospinal fluid shunting can be carried out if medicinal treatment is insufficient [7].

The treatment of acute vitamin A toxicity involves monitoring for indicators of intracranial hypertension, hydration, and stopping exposure. Acetazolamide may also have a direct impact on the metabolism of vitamin A [8].

## Conclusions

Benign intracranial hypertension constitutes an exclusion diagnosis, and imaging investigations should always be carried out to exclude other structural and obstructive abnormalities. Permanent vision loss and pain are the two main consequences of this illness. Various clinical and pathological disorders that can result from hypervitaminosis A may lead to significant morbidity and, in rare cases, mortality; therefore, its recognition is very crucial. The purpose of this case report was to raise awareness of the possibility that hypervitaminosis A can be the cause of benign intracranial hypertension in children.

## References

[1] Vollbracht R, Gilroy J (1976). Vitamin A induced benign intracranial hypertension. Can J Neurol Sci.

[2] Bhettay EM, Bakst CM (1988). Hypervitaminosis A causing benign intracranial hypertension. A case report. S Afr Med J.

[3] Malem A, Sheth T, Muthusamy B (2021). Paediatric idiopathic intracranial hypertension (IIH)-a review. Life (Basel).

[4] Balbi GG, Matas SL, Len CA, Fraga MM, Sousa IO, Terreri MT (2018). Pseudotumor cerebri in childhood and adolescence: data from a specialized service. Arq Neuropsiquiatr.

[5] Albakr A, Hamad MH, Alwadei AH (2016). Idiopathic intracranial hypertension in children: diagnostic and management approach. Sudan J Paediatr.

[6] Sharieff GQ, Hanten K (1996). Pseudotumor cerebri and hypercalcemia resulting from vitamin A toxicity. Ann Emerg Med.

[7] El-bouz M, Msaaf H, Aoued LL, Gueddari W (2022). Rare cause of pseudotumor cerebri in children. Pediatr Oncall.

[8] Lagacé M, Oskoui M, Myers K (2024). Not a benign vitamin: infant with vitamin a toxicity and acute intracranial hypertension. Can J Neurol Sci.

